# Recent Advances and Future Perspectives in Plant Auxin Biology

**DOI:** 10.3390/plants15050683

**Published:** 2026-02-25

**Authors:** Dong-Wei Di, Verena Kriechbaumer

**Affiliations:** 1 State Key Laboratory of Soil and Sustainable Agriculture, Institute of Soil Science, Chinese Academy of Sciences, Nanjing 210008, China; 2 University of Chinese Academy of Sciences, Beijing 100049, China; 3 School of Biological and Medical Sciences, Faculty of Health, Science and Technology, Oxford Brookes University, Oxford OX3 0BP, UK

## 1. Introduction

The phytohormone auxin, predominantly as indole-3-acetic acid (IAA), functions as a master regulator that orchestrates plant growth, development, and adaptation to environmental cues [1,2]. Its spatiotemporal distribution is fine-tuned by a multi-layered regulatory network encompassing biosynthesis, metabolism, polar transport, and signal transduction, thereby integrating key processes ranging from embryogenesis and organ patterning to stress resilience [3,4,5]. In the context of escalating global climate change and increasing frequency of abiotic stresses, deciphering how auxin-mediated networks equip plants with phenotypic plasticity and adaptive capacity is not only a fundamental scientific question but also a critical need for sustainable agriculture and ecosystem preservation [2].

In recent years, auxin biology has witnessed a profound conceptual transformation, shifting from the classical nuclear-centric SCF^TIR1/AFB^-Aux/IAA-ARF signaling pathway toward a more complex, decentralized, and multi-tiered regulatory system. Central to this paradigm is the emergence of a dual-channel signaling framework. At the nuclear level, TIR1/AFB receptors utilize their intrinsic adenylate cyclase activity to generate confined cAMP pools, which are essential for the precise transcriptional reprogramming of auxin-responsive genes [6,7,8,9]. Concurrently, at the plasma membrane, the ABP1/ABL–TMK module initiates rapid phosphorylation cascades that directly modify pivotal targets such as PIN-formed efflux carriers and H^+^-ATPases, thereby coordinating auxin transport and acid-growth responses within minutes [10,11,12,13,14,15,16]. This bipartite signaling model not only resolves the long-standing dichotomy between rapid physiological adjustments and delayed transcriptional changes but also illustrates how auxin signaling is temporally and spatially layered to optimize plant adaptation [9,10].

Expanding upon these mechanistic foundations, contemporary research has increasingly focused on the self-organizing properties of the auxin system. Through the integration of functional analyses of self-organizing transport modules with high-resolution structural studies of AUX1/LAX influx and PIN efflux carriers, fundamental biophysical principles governing the spatiotemporal patterning of auxin distribution are being unveiled [11,17,18,19,20]. These advances highlight how modularity, feedback loops, and physical constraints collectively shape auxin-mediated development and stress responses.

This Special Issue, titled “Advances in Plant Auxin Biology,” assembles a collection of cutting-edge reviews and original research articles that collectively examine the complexity and integrative nature of auxin regulatory networks. Contributions span multiple scales of analysis, from molecular enzymology and real-time ion/metabolite flux measurements to whole-plant phenology and polyploid genome regulation, thereby offering a cohesive and multi-dimensional perspective on auxin as a central orchestrator of plant life.

## 2. Foundational Insights from Comprehensive Reviews

This Special Issue integrates several foundational review articles that collectively refine and advance the theoretical framework of auxin biology. Gao et al. systematically synthesize the current understanding of the auxin regulatory system, offering a detailed elucidation of tryptophan-dependent IAA biosynthesis with a focus on the central IPyA pathway [21]. They further delineate key mechanisms governing IAA inactivation, including GH3-mediated amide conjugation and DAO-catalyzed oxidation. By integrating recent insights into polar auxin transport components—such as the PIN, AUX1/LAX, and ABCB protein families—the authors critically examine the layered complexity of auxin signaling, spanning from canonical TIR1/AFB-mediated transcriptional regulation to rapid non-transcriptional responses. Notably, the review emphasizes the context-dependent role of auxin in abiotic stress adaptation, illustrating how its signaling is often suppressed under drought or salinity, yet can be recruited to drive adaptive morphological responses such as hyponasty under heat stress.

In the context of auxin metabolic regulation, Luo et al. present a focused examination of the IAA–amino acid amide conjugation pathway mediated by the GRETCHEN HAGEN 3 (GH3) family [22]. Their review provides a comprehensive analysis of the enzymatic mechanisms, transcriptional control, and functional divergence of GH3 members across plant species. Building on established evidence, the authors systematically argue that GH3 enzymes extend beyond their classical role as mere “buffers” of IAA homeostasis; instead, they act as pivotal signaling hubs that integrate diverse environmental stress cues. These include drought, temperature variation, pathogen challenge, and nutrient imbalance, through which GH3s dynamically fine-tune active IAA pools. Furthermore, the review underscores that the development of GH3-specific chemical inhibitors represents a crucial experimental strategy for elucidating the functional redundancy and specificity inherent to this gene family.

Ma et al. [23] provide a systematic review of the evolving understanding of IAOx-dependent IAA biosynthesis within Brassicaceae, tracing its conceptual progression from an early linear model to a more complex and debated framework [24,25]. Recent genetic and metabolic evidence has challenged the established linear pathway model involving specific *CYP71A*, *NIT*, and *AMI* gene families [25]. Instead, IAOx is now viewed as a potential metabolic branch point whose flux towards IAA may involve yet-uncharacterized enzymatic routes, operating alongside or independently of its role in defense-related metabolites such as glucosinolates [25]. This ongoing reassessment highlights auxin biosynthesis, particularly via IAOx, as a potentially critical and underexplored interface in the plant’s strategic allocation of resources between growth and stress adaptation. By integrating recent molecular insights with persistent knowledge gaps, the authors propose several priority research trajectories, including identifying the enzyme(s) catalyzing the direct conversion of IAOx to IAA; clarifying the biosynthetic origins and signaling roles of intermediates IAN and IAM; elucidating how metabolic channeling and cell-type-specific regulation coordinate growth-defense trade-offs; and examining the evolutionary conservation and adaptive divergence of this pathway beyond Brassicaceae [26].

## 3. Polar Auxin Transport: The Architect of Form

The translation of hormonal gradients into morphological outcomes is orchestrated by the polar auxin transport (PAT) system. Wenzel et al. present robust experimental support for its conserved morphogenetic function across diverse dicot species. By pharmacologically inhibiting PAT in four taxonomically distinct dicots exhibiting varied leaf forms, including pinnate and palmate architectures—the authors establish that PAT is indispensable for establishing discrete auxin maxima, which in turn govern leaf complexity and the elaboration of venation networks [27]. Their findings, corroborated by computational simulations, demonstrate that PAT disruption leads to auxin distribution homogenization, ultimately driving leaf simplification and a convergence of venation toward parallelized patterning. Collectively, this study consolidates the role of PAT as an evolutionarily conserved and fundamental regulator of plant morphological architecture.

## 4. Context-Specific Regulation: Driving Unique Phenotypes Through Auxin

Auxin also plays a pivotal role in plant adaptation to specific physiological and environmental contexts. Mao et al. investigated the physiological basis of the unique year-round shooting phenomenon in the woody bamboo species *Cephalostachyum pingbianense* [28]. Through comprehensive annual hormone profiling, they demonstrated that sustained high levels of IAA and ABA in dormant rhizome buds, coupled with an elevated IAA/ABA ratio, are robustly correlated with shoot emergence. This indicates that the bamboo’s rhythmic growth is governed primarily by internal hormonal homeostasis rather than external seasonal cues, underscoring the significance of auxin homeostasis in shaping adaptive growth strategies.

At the interface of primary and specialized metabolism, Martin et al. revealed that targeted modulation of auxin signaling, via exogenous IAA or the transport inhibitor 2,3,5-Triiodobenzoic Acid (TIBA), can effectively reprogram alkaloid biosynthesis in *Annona emarginata* [29]. TIBA treatment notably enhanced alkaloid accumulation in roots, a response potentially driven by redirected photosynthate allocation. This aligns with earlier studies in other plant systems where auxin signaling has been shown to modulate the expression of key alkaloid biosynthetic genes (e.g., *TDC* in *Catharanthus roseus*) and influence precursor flux through shared pathways such as the shikimate–tryptophan route [30,31]. Thus, while the precise molecular mechanisms in Annona remain to be fully elucidated, the observed metabolic shifts suggest an interplay between auxin physiology and secondary metabolism, highlighting the potential of hormonal intervention as a strategic tool for the targeted production of valuable plant specialized metabolites.

## 5. Allelic Bias in Polyploids: Reshaping the Auxin Response Network

In polyploid crops, the contribution of homoeologous alleles introduces an additional layer of regulatory complexity. Zhao et al., while investigating embryogenic callus formation in ABB banana, uncovered striking allele-specific dominance within the auxin signaling pathway [32]. Their study revealed that A-genome-derived alleles of key transcription factors and signaling components overwhelmingly dominate transcriptional reprogramming, thereby directing cell fate transitions. This work elucidates how genomic architecture and allelic bias can fundamentally reshape the output of the core auxin response network in polyploid systems.

## 6. Empowering Auxin Physiology Through Advanced Technologies: NMT as a Key Tool for Real-Time Flux Analysis

Dissecting the rapid physiological functions of auxin necessitates the use of advanced technological platforms. Zhang et al. examine the essential contribution of Non-invasive Micro-test Technology (NMT) to the analysis of plant abiotic stress responses [33]. Capable of real-time, in situ, and high-sensitivity detection of transmembrane ion and molecule fluxes, such as IAA, H^+^, Ca^2+^, and K^+^, NMT provides a powerful tool for resolving the spatiotemporal dynamics of auxin under stress. The authors emphasize that NMT not only directly measures IAA transport across membranes but also concurrently tracks its coordination with other signaling pathways, including Ca^2+^ signaling and H^+^ flux [34,35]. This multimodal capability enables a systems-level understanding of how auxin distribution and activity are regulated during stress adaptation, influencing processes such as stomatal conductance, root system reorganization, and ion homeostasis. Collectively, these insights position NMT as a pivotal methodological bridge that integrates auxin-related molecular mechanisms with organism-level physiology, offering a vital physiological dimension to molecular investigations of auxin-dependent stress responses.

## 7. Outlook

Over the past decade, auxin research has undergone profound paradigm shifts, transitioning from a singular focus on nuclear receptor mechanisms to the recognition of a sophisticated cell surface co-receptor system. This evolution in perspective extends from earlier models of linear biosynthetic pathways toward an integrated understanding of complex metabolic networks, and from static descriptions of hormone distribution to the exploration of dynamic, self-organizing patterning processes. Collectively, these advances reveal a signaling landscape that is far more intricate, context-dependent, and dynamically regulated than previously appreciated. This refined framework not only redefines our understanding of auxin-mediated development but also highlights the multi-layered nature of hormone signaling in plants. At this new conceptual frontier, the field is poised to address critical challenges in systemic integration, particularly in elucidating how spatially and temporally distinct signaling modules are coordinated across scales.

(i)  Elucidating unknown molecular and biochemical mechanisms

Although the core framework of auxin signaling has been largely delineated, critical gaps persist in our understanding of key molecular and biochemical mechanisms. A primary research priority lies in resolving the full-length three-dimensional structures of classical long-loop PIN family proteins (e.g., PIN1), which would enable atomic-level insights into the structural basis of their autoinhibition, kinase-dependent activation, and polar localization. Furthermore, it is essential to comprehensively elucidate the functional network of cAMP produced by TIR1/AFB receptors as a second messenger, including the identification of its direct downstream targets and potential plant-specific cyclic nucleotide effector proteins. Additionally, an integrated approach combining genetics, metabolomics, and other advanced methodologies is urgently needed to uncover the yet-uncharacterized biochemical pathways underlying established phenotypes, such as the precise origin and regulatory nodes of excess auxin synthesis in *superroot 2* (*sur2*) mutants. The elucidation of these molecular and biochemical mechanisms will not only provide a robust foundation for constructing predictive and testable theoretical models but also facilitate the transition of auxin signaling research from qualitative description toward a quantitative systems biology paradigm.

(ii)  Deciphering spatiotemporal specificity and evolutionary adaptability of auxin signaling pathways

Auxin signal transduction is not a fixed, rigid pathway but exhibits remarkable context dependency and evolutionary plasticity. For instance, the TMK signaling module performs antagonistic functions in roots and hypocotyls, while the IAOx biosynthetic pathway has evolved a new role in defense responses within Brassicaceae. Future research should systematically map the activity dynamics and interaction networks of various signaling modules across different cell types, developmental stages, and environmental conditions with higher spatiotemporal resolution. Concurrently, moving beyond model plant studies, comparative genomics and evolutionary developmental biology approaches should be employed to trace the innovation and evolution of core components involved in auxin perception, signal transduction, and transport during the adaptive radiation of land plants. This will help address a fundamental scientific question: how can a relatively conserved hormonal system be “reprogrammed” through regulatory mechanisms to support the vast diversity of plant morphogenesis?

Collectively, recent findings reinforce the view that auxin-mediated development emerges from self-organizing systems integrating local biosynthesis, directional transport, and dynamic signaling feedback. The interplay between nuclear and non-nuclear auxin signaling pathways, coupled with interactions with other hormonal networks, enables plants to generate robust yet flexible developmental patterns. Future research aimed at linking molecular mechanisms to emergent properties across spatial and temporal scales will be crucial for fully understanding how auxin orchestrates plant form and function. In-depth exploration of these questions will not only reveal fundamental principles of plant life but also provide important theoretical insights and technical inspiration for sustainable agricultural development and biomaterial innovation.

## Data Availability

No new data were created or analyzed in this study. Data sharing is not applicable to this article.
